# Cardiovascular Safety Profile of Romosozumab: A Pharmacovigilance Analysis of the US Food and Drug Administration Adverse Event Reporting System (FAERS)

**DOI:** 10.3390/jcm10081660

**Published:** 2021-04-13

**Authors:** Annika Vestergaard Kvist, Junaid Faruque, Enriqueta Vallejo-Yagüe, Stefan Weiler, Elizabeth M. Winter, Andrea M. Burden

**Affiliations:** 1 Department of Chemistry and Applied Biosciences, Institute of Pharmaceutical Sciences, ETH Zurich, 8092 Zurich, Switzerland; annika.kvist@pharma.ethz.ch (A.V.K.); enriqueta.vallejo@pharma.ethz.ch (E.V.-Y.); stefan.weiler@toxinfo.ch (S.W.); 2 Leslie Dan Faculty of Pharmacy, University of Toronto, Toronto, ON M5S 3M2, Canada; junaid.faruque@mail.utoronto.ca; 3 National Poisons Information Centre, Tox Info Suisse, Associated Institute of the University of Zurich, 8032 Zurich, Switzerland; 4 Center for Bone Quality, Department of Internal Medicine, Division of Endocrinology, Leiden University Medical Center, 2333 ZA Leiden, The Netherlands; e.m.winter@lumc.nl

**Keywords:** osteoporosis, romosozumab, safety, pharmacovigilance

## Abstract

**Background**: Cardiovascular safety concerns for major cardiovascular events (MACE) were raised during the clinical trials of romosozumab. We aimed to evaluate the cardiovascular safety profile of romosozumab in a large pharmacovigilance database. **Methods:** All cases reported between January 2019 and December 2020 where romosozumab was reported were extracted from the Food and Drug Administration Adverse Event Reporting System (FAERS). The outcome of interest was MACE (myocardial infarction (MI), stroke, or cardiovascular death). A disproportionality analysis was conducted by estimating the reporting odds ratios (RORs) and 95% confidence intervals. Disproportionality analyses were stratified by sex and reporting region (US, Japan, other). **Results:** Of the 1995 eligible cases with romosozumab, the majority (*N* = 1188; 59.5%) originated from Japan. Overall, 206 suspected MACE reports were identified, of which the majority (n = 164; 13.8%) were from Japan, and 41 (5.2%) were from the United States (US). Among Japanese reports, patients were older and more frequently male than reports from the US. Similarly, cases with a reported MACE were older and had higher reports of cardioprotective drugs than those without cardiovascular events. Elevated reports for MACE (ROR 4.07, 95% CI: 2.39–6.93) was identified overall, which was primarily driven by the significant disproportionality measures in the Japanese reports. **Conclusions:** The current pharmacovigilance study identified a potential signal for elevated MACE, particularly in Japan. The results support the current safety warnings from the Food and Drug Administration (FDA) and the European Medicines Agency (EMA) to avoid use in high-risk patients.

## 1. Introduction

Romosozumab is a monoclonal antibody approved for patients (predominantly postmenopausal women) with a high risk of fracture. The clinical efficacy and safety of romosozumab were demonstrated in the two pivotal Phase 3 randomized controlled trials (RCTs), the active-controlled fracture study in postmenopausal women with osteoporosis (ARCH) and the fracture study in postmenopausal women with osteoporosis (FRAME) [1,2]. In both trials, romosozumab significantly reduced the incidence of new vertebral fractures at 12- and 24-months. Due to evidence of high efficacy, romosozumab was first approved by the Japanese Pharmaceutical and Medical Devices Agency (PMDA) in January 2019 for osteoporosis patients (both men and women) with a high risk of fracture [3]. However, the United States (US) Food and Drug Administration (FDA), Health Canada, and the European Medicines Agency (EMA) denied the initial application for approval citing concerns of cardiovascular safety [4,5,6]. However, the FDA, Health Canada, and the EMA approved romosozumab in April, June, and December 2019, respectively, following further review of the ARCH trial data [7,8,9]. All agencies, except the PMDA, included boxed warnings at the time of approval explicitly stating the elevated cardiovascular risk. Additionally, the EMA included a specific statement for the contraindication in patients with either a history of a major cardiovascular event [7,8] or a history of stroke or myocardial infarction (MI) [9]. A safety warning for the risk of cardiovascular events was only recommended by the PMDA following a review of post-market case studies in September 2019 [10,11]. To date, romosozumab approval is largely limited to postmenopausal women, except in Japan, where romosozumab is also approved for men.

The preliminary safety information for romosozumab stemmed largely from the ARCH and FRAME trials [1,2]. Individually, the occurrence of death, major cardiovascular event (MACE) (composite of MI, stroke, and cardiovascular death), osteonecrosis of the jaw (ONJ), acute atypical femoral fracture (AFF), and hypocalcemia were elevated in the treatment arms of the ARCH and FRAME trials [1,2]. A post hoc analysis of MACE in both trials identified an imbalance in the observed events between the romosozumab and control arms; however, only the ARCH trial found a statistically significant result in the post hoc analysis (hazard ratio (HR) 1.87 (95% confidence interval (CI) 1.10–3.14) [4,12]. Additionally, a smaller placebo-controlled study in men (the BRIDGE trial) found higher cardiovascular events, such as cardiac ischemic and cerebrovascular events, in the romosozumab arm compared to placebo [13]. However, this could be explained by a slightly elevated baseline risk for cardiovascular events (77.3% in the romosozumab arm vs. 71.6% in the placebo arm), yet fewer subjects on cardioprotective medications (57.1% in the romosozumab arm vs. 61.7% in the placebo arm) [13]. The lack of cardioprotective drugs in the presence of other cardiovascular risk factors may partially explain the elevated risk observed in this trial. It is notable that Japanese patients were not included in the ARCH trial, and the majority of evidence regarding the safety in Japanese patients was based on a subgroup analysis of 187 Japanese patients in the FRAME trial where no significant difference in cardiovascular events was observed [14,15].

Despite impressive anti-fracture effects, the safety of romosozumab remains an important and highly debated topic [16,17,18,19]. Thus, large real-world post-marketing evidence is urgently needed. With this in mind, we aimed to evaluate for the first time the adverse events reported to the US FDA Adverse Event Reporting System (FAERS) for romosozumab, with a focus on serious cardiovascular events, covering the entire period since first approval.

## 2. Methods

### 2.1. Data Source

The US FAERS (or FDA AERS) database is a publicly available database of all spontaneous adverse event reports that are submitted to the US FDA and includes over 20 million reports [20]. FAERS includes real-world data of suspected adverse drug reactions (ADRs) that are reported to the FDA via the MedWatch program. While the majority of reports originate from the US, approximately one-third of the annual reports are from non-US (foreign) countries [21]. Reports can be submitted by healthcare professionals, consumers, and manufacturers globally. While reports by healthcare professionals and consumers are voluntary, all suspected ADRs received by marketing authorization holders regarding a product are required to be submitted to the FDA. Similar pharmacovigilance databases exist, including the European Union (EU) database EudraVigilance [22], as well as the World Health Organization (WHO) VigiBase [23], which contains all case safety reports submitted by WHO member countries [24]. While the geographical region of focus is different between these databases, with FAERS predominantly covering the US, EudraVigilance covering the European Union, and VigiBase with a global focus, previous studies have identified overlapping results [24,25].

Quarterly data of all adverse events reported to the FDA are available for public download [26]. Information includes case demographics (age, sex), suspect drug(s), concomitant or interacting drug(s), outcome(s) or events(s), reporting country, and reporter type. The suspected adverse events in the FAERS database are recorded according to the Medical Dictionary for Regulatory Activities (MedDRA^®^). While the safety reports provide information on the patients at the time of the adverse event, a complete medical history is not available. Thus, with this type of data, it is not possible to determine if the patients had relevant comorbidities that may predispose them to the outcomes of interest. To some extent, this information can be identified based on the reported co-medications. However, as the reports in FAERS are generated through passive (voluntary) surveillance, the level of completeness of the information provided can vary. For example, while some reports may contain complete information on all concomitant medications used, particularly those identified as potential suspect drugs, at the time of the adverse event, others may only list the drug(s) believed to be the likely cause of the adverse event. Thus, it can be challenging to identify the baseline cardiovascular risk of cases based on their reported medication history. Additionally, while the timing of the drug start and adverse event data can be entered, the reliability of this information is poor and therefore not considered in this study.

### 2.2. Study Population

All quarterly (Q) data from FAERS between 1 January 2019 (Q1 2019; first approval period) and 31 December 2020 (Q4 2020; latest FAERs update) were extracted. From these cases, all reports where romosozumab was reported were identified. Cases where the FDA report date was before 1 January 2019 were excluded. Patient age was reviewed, and illogical values were transformed to “unknown” values.

### 2.3. Outcomes of Interest

The events of interest were identified using specific MedDRA^®^ preferred terms (PTs) within the following high-level terms (HLTs): “cardiac disorders”, “nervous system disorders”, and “vascular disorders”. An overview of the specific PTs selected can be identified in the Supplementary Table S1. The primary outcome of interest was a major cardiovascular event (MACE: myocardial infarction, stroke, or cardiac death). As a secondary analysis, we included other cardiovascular events (bleeding, thrombosis, general cardiovascular events (coronary artery disease or cardiac disorder)).

### 2.4. Statistical Analysis

Demographic information was summarized overall and stratified by region of the report and cardiovascular outcomes (MACE, other cardiovascular, or any (MACE or other)). In addition to summarizing the case characteristics and outcomes, we identified the frequency with which other cardiovascular medications were recorded as either suspect, interacting, or concomitant drugs to provide an indicator of potential baseline risk. Cardiovascular medications were identified using the active product ingredients (Supplementary Table S2) and categorized as anticoagulants, antiplatelets, angiotensin converting enzyme inhibitors (ACEi), angiotensin receptor blockers (ARBs), beta-blockers, and calcium channel blockers (CCBs). Individual counts with a reported frequency of less than five were compressed for patient privacy protection. Differences between groups (MACE vs. no cardiovascular outcome and the US vs. Japan) were tested for statistical significance using chi square with Yates’s correction or *t*-tests, as appropriate, using *p* < 0.05 as the significance level.

A disproportionality analysis was conducted by estimating the reporting odds ratios (RORs) and 95% confidence intervals (CI) and the information component (IC) with the corresponding lower bound 95% credibility interval (IC025) [27]. The measures of disproportionality compare the observed versus expected reporting ratio for a specific event and medication. The ROR and IC use a case–non-case approach to compare the observed versus expected reporting ratio for an event and medication of interest. For interpretation, a signal of disproportionate reporting was considered when the lower-bound 95% CI of the ROR was above 1.0 and an IC025 above 0 [28,29]. The expected reporting ratio was calculated using all other drug/event reports in the FAERS data between 1 January 2019 and 31 December 2020. An overview of the equations for these calculations can be found in Appendix A. Outcomes used in the disproportionality analysis were: MACE, other cardiovascular events, myocardial infarction, stroke, cardiovascular death, bleeding, or thrombosis. We conducted disproportionality analyses for all outcomes of interest overall and stratified by region of reporting.

In secondary analyses, we examined patient characteristics and the disproportionality analysis stratified by sex. Additionally, we examined the number of reports for MACE in Japan by month to examine time trends following the 2019 post-market investigation and subsequent addition of the cardiovascular warning in the package insert. Finally, we conducted the disproportionality analysis in those aged 50 years or older (those with missing age were excluded from this analysis). This analysis was carried out as the mean age of those receiving romosozumab is expected to be postmenopausal, and this will restrict the base comparator population FAERS to a more similar age group. Analyses and graphics were conducted using R statistical [30] software (version 4.0.3) and GraphPad Prism 8 [31].

## 3. Results

**Figure 1:**
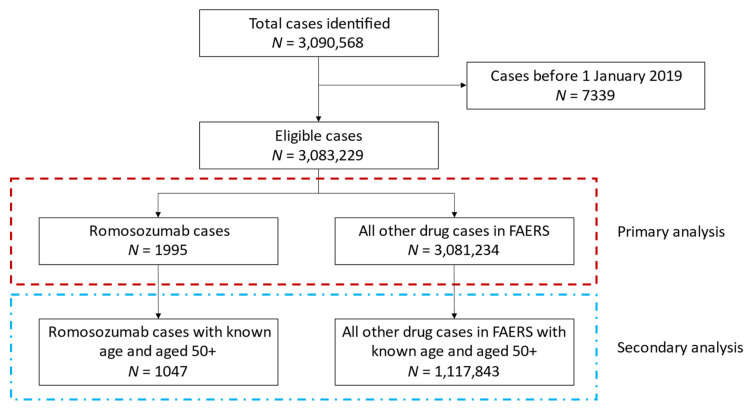
Study flow diagram for included cases identified from quarterly files in the US Food and Drug Administration Adverse Event Reporting System (FAERS) between 1 January 2019 and 31 December 2020.

A total of 3,083,229 eligible cases were identified from the quarterly files between 1 January 2019 and 31 December 2020 from FAERS (Figure 1). Of which, 1995 case reports included romosozumab as a reported drug.

Table 1 summarizes the demographic characteristics of the included cases, stratified by the region of report origin (the US vs. Japan). Overall, the majority of the reported cases were females (76.1%), while 8.9% were males, and 15.0% had no sex reported. The mean age of cases was 77.0 years (standard deviation (sd) 10.2). More than half of the identified reports originated from Japan (59.5%). Reports from Japan were more often male (13.0% vs. 2.8%, *p* < 0.05), were older (mean age 80.2 (sd 9.5) vs. 71.7 (sd 8.5), *p* < 0.05), and had more serious outcomes, including death (13.3% vs. 2.3%, *p* < 0.05) or hospitalization or intervention (49.6% vs. 8.5%, *p* < 0.05), when compared to reports from the US, respectively. Among the outcomes of interest, there were higher reports of MACE from Japan (13.8%) compared to the US (5.2%, *p* < 0.05), which was primarily driven by elevated reports of cardiovascular death in Japan (7.0%) compared to the US, where less than five events were observed. There were significantly elevated reports of cardiovascular medications between Japan and the US, in particular for CCBs (7.7% vs. 1.0%) and antiplatelets (4.0% vs. 1.5%).

Table 2 presents the patient demographics stratified by the cardiovascular outcome of interest. Overall, there were 1740 cases without a cardiovascular outcome reported and 206 with a MACE, 58 with another cardiovascular event, and 255 with any cardiovascular outcome. The mean age of patients with MACE (81.2 years, sd 9.5) was significantly older than those without a cardiovascular event (76.4 years, sd 9.9, *p* < 0.05), and the proportion aged 80+ among cases with a MACE (47.6%) was higher compared to those without (19.3%, *p* < 0.05). The majority of MACE reports were from Japan (79.6%). There were significant differences in the severity of outcomes between cases with a reported MACE compared to non-cardiovascular events. In particular, compared to those without a cardiovascular event, those with a reported MACE were more likely to have a fatal outcome (26.2% vs. 6.7%, *p* < 0.05). The proportion of fatal events was also higher among those with a MACE compared to those with other cardiovascular events (26.2% vs. 12.1%, *p* < 0.05). Patients with MACE events were more likely to have a cardiovascular medication co-reported. There were significantly higher reports of CCBs (16.5% vs. 3.4%), antiplatelets (10.7% vs. 2.0%), ARBs (10.7% vs. 2.3%), beta-blockers (9.7% vs. 1.4%), and anticoagulants (6.3% vs. 1.2%) among those with a MACE compared to those without, respectively.

The analysis stratified by sex is provided in Supplementary Table S3. On average, males were older, with a mean age of 79.4 years (sd 9.4) compared to females (mean 76.7, sd 10.1, *p* < 0.05). Among the male cases (*N* = 177), the majority were from Japan (*N* = 154, 87.0%). The proportion of fatal events was significantly higher among males (20.3%) than females (9.2%, *p* < 0.05). MACE events were more frequently reported among males (16.9%) compared to females (10.5%, *p* < 0.05), which was driven by significant differences in cardiovascular death (7.9% males vs. 4.2% females) and MI (5.6% males vs. 1.9% females). Additionally, males had higher reported use of antiplatelets (7.3% vs. 3.0%), beta-blockers (5.6% vs. 2.4%), and CCBs (10.2% vs. 5.3%), compared to women.

The number of MACE reports from Japan, plotted by month, is provided in (Supplementary Figure S1). There was a spike in the number of reports provided to the FDA in September 2019 (*N* = 28), which corresponds to the date of the PMDA investigation and subsequent modification to the package-insert safety warning. However, no noticeable time-trend was observed.

In the disproportionality analyses, the expected reporting ratios for the outcomes of interest were calculated using every other eligible case in FAERS that did not include romosozumab (*N* = 3,081,234, Figure 1). The ROR and IC025 values for all outcomes are provided in Table 3. Disproportionate reporting for the composite endpoint MACE was observed (ROR 4.07, 95% CI: 2.39–6.93; IC025 1.67). Similarly, all individual MACE outcomes showed elevated disproportionate reporting: MI (ROR 3.10, 95% CI: 1.43–6.72; IC025 1.06), stroke (ROR 3.47, 95% CI: 1.81–6.69; IC025 1.37), and cardiovascular death (ROR 4.90, 95% CI: 2.55–9.40; IC025 1.85). We did not identify disproportionate reporting for other cardiovascular events. The results from the sensitivity analysis, restricting the population to those aged 50+, were highly similar to the primary results (Supplementary Table S4).

**Figure 2:**
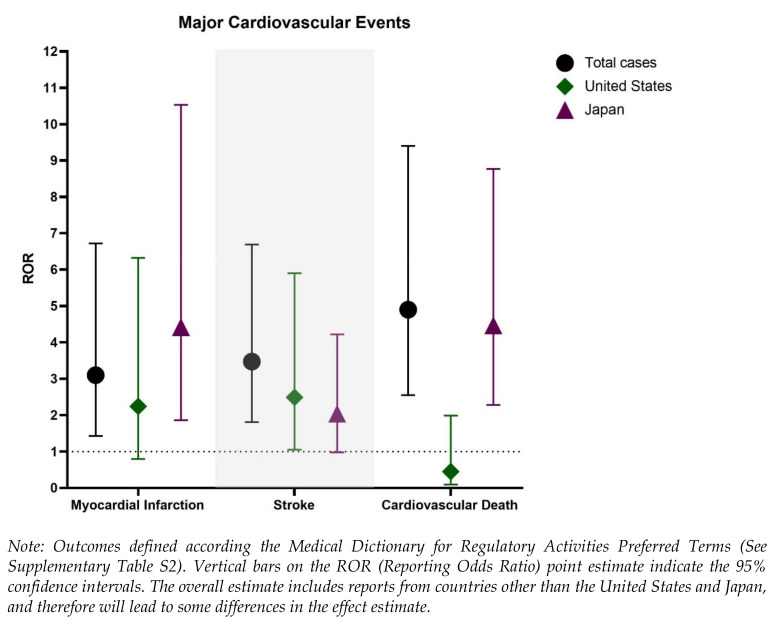
Disproportionality analysis of suspected major cardiovascular events (MACE) for romosozumab compared with all other medications in the US Food and Drug Administration Adverse Event Reporting System (FAERS), stratified by the overall (*N* = 1995) and reporting region (United States, *N* = 787) and Japan (*N* = 1188).

In Supplementary Table S5, we provide the results of the disproportionality analysis for all outcomes, stratified by sex (males and females) and region of reporting (the US and Japan). The trends were similar for MACE events, with significant signals based on the IC025 identified in all strata. However, the confidence interval for the ROR among reports originating from the US was not significant (ROR 1.83, 95% CI: 0.80–4.00). The results were similar when assessing the individual MACE events for myocardial infarction and cardiovascular death, with the US revealing non-significant RORs. However, for stroke, all strata revealed significant and elevated measures of disproportionality. Figure 2 illustrates the RORs and 95% CIs for the individual MACE events, stratified by sex and region of reporting.

## 4. Discussion

Following the cardiovascular safety concerns raised during the clinical development of romosozumab, we performed a pharmacovigilance study in FAERS to study the events of interest in post-marketing safety reports. In this pharmacovigilance study, 59.5% of cases were reported from Japan. Of the 206 reported MACE outcomes, 164 (79.6%) originated from Japan. Those with a reported MACE were older and more likely to report the use of other cardiovascular medications—such as CCBs, ARBs, and antiplatelets—suggesting a potential elevated cardiovascular risk profile. The disproportionality analysis identified elevated reports for MACE (including MI, stroke, and cardiovascular death) but not for other cardiovascular events. More than half of the reports in the FAERS database originated from Japan, which was the first country to authorize the use of romosozumab and did not initially include a safety warning for cardiovascular risk. Additionally, the majority of reports related to male cases (87%) were from Japan, where approval was not restricted to postmenopausal women.

Our results identified higher than expected reports for the adjudicated outcome for MACE, including the individual events of MI, stroke, and cardiovascular events with romosozumab compared to all other cases in the FAERs database. Our results are in-line with the current safety concerns of serious cardiovascular risk raised from the ARCH and BRIDGE clinical trials and early post-market data from Japan [1,13]. However, in contrast, a meta-analysis comparing adverse events across six trials, stratified by a control group, did not identify any significant difference in the incidence of the composite endpoint of all cardiovascular safety between the treatment groups and therefore did not stratify by type of cardiovascular event [32]. In our analysis, we observed that other suspected cardiovascular events, such as thrombosis and bleeding, were non-significant, and differences were observed by region, with the US reports not showing elevated reports of the individual MACE events. Similarly, a second meta-analysis by Kaveh et al., focusing on serious cardiovascular events and cardiovascular death, found no statistical differences between romosozumab and a placebo [17]. However, the analysis was restricted to data from the FRAME [2] and BRIDGE [13] trials and therefore did not include the ARCH trial [1], which was the primary trial showing an elevated incidence of MACE.

Our results highlighted differences between reports originating from Japan compared to the US or other countries. The cases reported from Japan were older, more often including males, had higher fatal outcomes, and more frequently reported MACE as a suspected outcome, compared to reports from the US. Additionally, we also observed higher reports of cardioprotective drugs—particularly CCBs and antiplatelets—in the reports from Japan. While there may be a number of reasons for this, and the overall prevalence is low, this may be explained by the later addition of the cardiovascular safety warnings to the product information in Japan in comparison to the other studied countries [10,11,14]. Thus, as noted by Kawaguchi [14], it is possible that romosozumab may have been used in a higher-risk population in Japan until September 2019, when the warnings were placed. However, we note that we did not observe a clear trend in the number of MACE reports pre- and post-September 2019. Following the PMDA investigation, the Japanese package inserts were revised to include precautions and warnings for cardiovascular risk. Thus, while it is recommended that patients with a history of ischaemic heart disease or cardiovascular disorder in the prior year should not receive romosozumab, the warning states that administration to higher-risk patients should be determined after weighing the benefits of reducing fracture risk and the onset of cardiovascular events [11]. Consequently, it is unclear if this warning would lead to a substantial change in the prescribing pattern.

Additionally, we observed that the majority of males were from Japan, and the proportion of cardiovascular events were elevated among males. This may be a consequence of romosozumab being approved for both men and women by the PMDA, but only for women by the FDA, the EMA, and Health Canada. Previous evidence of romosozumab in men is limited to the BRIDGE trial, which included 55 to 90 year-old men with osteoporosis [13]. While the BRIDGE trial found an increase in adjudicated serious cardiovascular events, it was concluded that this could be due to an elevated baseline cardiovascular risk in the romosozumab group [13]. In our analysis, we observed higher reports of MACE among males, and evidence of higher baseline risk, with male cases being older and reporting more cardioprotective medications compared to women. Yet, the disproportionality analysis was not significantly different when comparing males and females in the FAERS database, with significant disproportionate reporting for all MACE outcomes observed in both sexes. Thus, it is possible that the elevated proportion of reported MACE in males was due to an elevated baseline risk (prevalence) for cardiovascular events when compared to women [33]. Nevertheless, further research on the safety of romosozumab in men is urgently needed to further understand if romosozumab may be a safe treatment option in this often understudied patient population.

To date, there is no known biological mechanism of action that may directly explain the elevated cardiovascular risk observed with romosozumab. However, as sclerostin may inhibit the formation of calcified plaques and therefore be cardio-protective [34], the romosozumab-induced inhibition of sclerostin could modulate Wnt signaling, resulting in vascular calcification, and in particular, atherosclerosis. However, a recent review of the evidence identified that romosozumab did not have a meaningful effect on the cardiovascular function in monkeys, nor did it lead to significant morphological changes in the cardiovascular calcification in the absence or presence of atherosclerosis. Therefore, it remains unclear if the observed cardiovascular risk is a result of an on-target effect of sclerostin or residual confounding. Moreover, specific alterations of pharmacokinetic, pharmacodynamic, or pharmacogenetic parameters of romosozumab in certain populations, such as the Japanese, have not been fully investigated. Without robust evidence, it is possible the risk observed in the ARCH trial was largely due to chance, as previously identified by Cummings and colleagues [16]. The baseline risk (absolute number of events) was very low in both the ARCH and FRAME trials, and it is therefore likely that neither trial was powered to detect a significant difference, thereby supporting the potential of a chance finding. However, the observed higher than expected reports in patients treated with romosozumab, along with the findings from RCTs, suggest that the cardiovascular risk of romosozumab requires further investigation.

Overall, the results from this large pharmacovigilance analysis support the current safety warnings put in place by the FDA, EMA, and Health Canada. The study findings further suggest a potentially higher risk of MACE among those with pre-existing risk factors for cardiovascular outcomes. In particular, the elderly (particularly those over the age of 80) and patients with co-reported cardiovascular medications. Thus, our results support that those patients with a history of, or are at risk of, cardiovascular disease and stroke should not be considered for treatment with romosozumab in daily clinical practice. Moreover, further evidence is required to elucidate the risk–benefit ratio in men.

### Strengths and Limitations

During the first years of new drugs in the clinic, pharmacovigilance studies are key to identify potential safety signals. This may be considered especially relevant to investigate or keep close attention to potential safety concerns triggered during the drug development phases prior to authorization. Thus, following the cardiovascular concerns observed in romosozumab clinical trials, it was of interest to study the safety reports for romosozumab, with special attention to cardiovascular events. To the best of our knowledge, this is the largest pharmacovigilance study assessing the cardiovascular safety of romosozumab and provides evidence from the first two years of real-world clinical use. Additionally, the observed higher reports of cardiovascular events for romosozumab in Japanese reports versus those from the US suggest that the FDA warnings restricting the use of romosozumab in patients with prior high cardiovascular risk, or the limited authorization for its use in women, may have had positive impact on reducing related safety events.

However, there are important limitations inherent to the use of pharmacovigilance data that should be acknowledged when interpreting the results, including reporting bias, causality assessment, and confounding. First, due to the nature of the FAERS data, being a spontaneous reporting system, we must be mindful of potential reporting bias that may occur due to differences in adverse event reporting practices across regions. For example, when a new medication includes a safety warning for a specific event (e.g., MACE), it is possible that (1) healthcare professionals will be more alert to the possibility of the outcome and report every case due to their awareness (overreporting), or (2) healthcare professionals will not report the event because it is already known, and pharmacovigilance systems are designed to identify new safety signals (underreporting). It is not possible to know from our data if either, or both, scenarios occurred. However, this reporting bias should be considered, particularly due to the differences in product label warnings between Japan and the other countries (including the US). Thus, while the elevated reporting in Japan may be a result of prescribing to high-risk patients due to the lack of an initial safety warning, or an unknown predisposing genetic risk factor, we cannot rule out the potential for selective reporting in our database. When assessing the time-trends in MACE reporting, we could see that the number of reports significantly increased in September 2019. This could be due to increased awareness of the cardiovascular risk associated with romosozumab. In the US reports, the insignificant disproportionality measures could be due to underreporting as the safety concern was known or a result of differences in reporting practices between the US and Japan. In the US, all case safety reports must be reported to the FDA, regardless of seriousness, while in Japan, it is only mandatory to report serious cases [35]. Additionally, there is no requirement for reported events to meet a threshold of plausibility (i.e., that the drug was the likely cause of the adverse event) in the US, which is not the case in Japan. Thus, while we would not expect to find major differences in the MACE cases reported, it is possible that the comparator groups in the country-specific disproportionality analysis may differ. Thus, further real-world evidence from large population-based electronic health records or administrative claims data is needed to overcome this limitation.

Second, causality cannot be inferred or determined from our disproportionality analysis as we do not have complete information on the patient history or the timing of the reported medication use and event of interest, and therefore cannot assess the temporality of events or adjust for relevant confounders. While a causality assessment may be carried out by some reporters prior to submitting cases to FAERS, we note that reports may come from a variety of sources, and therefore we cannot conclude that the causality of a drug–outcome relationship has been assessed equally in all cases. As such, the ROR and IC025 in this study should be viewed with caution and interpreted as evidence of higher than expected reporting and not necessarily association with higher risk [28].

Finally, due to the nature of the FAERS data, we cannot assess the potential for the outcome to be due to the drug or underlying confounding factors, which is essential when drawing causal associations. A previous meta-analysis combining the FRAME and ARCH data found that the hazard ratio for MACE was non-significant after adjusting for confounders (HR 0.55, 95% CI 0.27–1.14) [36]. In this study, we identified that patients with a suspected MACE were significantly older than those reporting other adverse events and more frequently reported the use of cardioprotective medications that suggest an elevated baseline risk. It is, therefore, possible that these cases may have had experienced a MACE without the use of romosozumab. Additionally, it has previously been observed that postmenopausal women with osteoporosis have a four-fold increased risk for cardiovascular events compared to postmenopausal women without osteoporosis [37]. In our disproportionality analysis, all cases without romosozumab reported to FAERS during the study period were used as the comparator. While these are not healthy controls, as they are cases with an ADR, confounding cannot be ruled out. In our secondary analysis, we restricted our disproportionality analysis to those aged 50 years or older to minimize my confounding by age and found consistent results. However, further work that aims to elucidate if there is a causal relationship between romosozumab and MACE must consider the potential for selection bias and confounding that is inherent with newly marketed drugs [38].

## 5. Conclusions

We identified higher than expected reports of MACE, which was particularly elevated in reports originating from Japan. The differences between the findings among Japanese reports and those from the United S may solely be the consequence of the delayed cardiovascular safety warnings, the approval for use in men, or the differences in reporting standards in Japan. However, since previous clinical evidence (from the two largest trials, FRAME, and ARCH) only included women, of which 10% were from Asia Pacific, further evidence in underrepresented populations is urgently needed. Moreover, while we cannot infer a causal risk due to the inherent limitations with pharmacovigilance data, the evidence does not dismiss concerns and calls for further real-world investigation. It is now imperative that further high-quality, real-world evidence that accounts for sources of bias and confounding is generated. Until such evidence is available, our results support the restricted prescribing recommendations in the boxed warnings that patients at a high risk of cardiovascular disease and stroke should not be considered for treatment with romosozumab.

## Figures and Tables

**Table 1:** Demographic characteristics of individual case safety reports with romosozumab, overall (*N* = 1995), and stratified by region of report (the United States, *N* = 787) and Japan (*N* = 1188).

	Total	United States	Japan
	1995	787 (39.4%)	1188 (59.5%)
Sex			
Female	1518 (76.1%)	833 (70.1%) †	16 (80.0%)
Male	177 (8.9%)	154 (13.0%) †	<5
Unknown	300 (15.0%)	201 (16.9%)	<5
Age			
Mean (SD)	77.0 (10.2)	71.7 (8.5)	80.2 (9.5) †
18–59	46 (2.3%)	29 (3.7%)	16 (1.4%) †
60–69	175 (16.6%)	117 (14.9%)	55 (4.6%) †
70–79	385 (36.4%)	167 (21.2%)	211 (17.8%)
80+	451 (42.7%)	70 (8.9%)	380 (32.0%) †
Unknown age	938 (47.0%)	404 (51.3%)	526 (44.3%) †
Seriousness Criteria			
Death	176 (8.8%)	18 (2.3%)	158 (13.3%) †
Hospitalized or Required Intervention	660 (33.1%)	67 (8.5%)	589 (49.6%) †
Life Threatening	48 (2.4%)	6 (0.8%)	41 (3.5%) †
Disabled	22 (1.1%)	6 (0.8%)	16 (1.3%)
Outcomes of Interest			
Major Cardiovascular Event	206 (10.3%)	41 (5.2%)	164 (13.8%) †
Myocardial Infarction	42 (2.1%)	13 (1.7%)	28 (2.4%)
Stroke	84 (4.2%)	27 (3.4%)	57 (4.8%)
Cardiovascular Death	86 (4.3%)	<5	83 (7.0%) †
Other Cardiovascular Event	58 (2.9%)	16 (2.0%)	42 (3.5%)
General cardiac events	16 (0.8%)	6 (0.8%)	10 (0.8%)
Bleeding	19 (1.0%)	--	19 (1.6%)
Thrombosis	23 (1.2%)	10 (1.3%)	13 (1.1%)
Other reported cardiovascular drugs			
Anticoagulants	38 (1.9%)	9 (1.1%)	29 (2.4%)
Antiplatelets	60 (3.0%)	12 (1.5%)	47 (4.0%) †
ACE inhibitors	14 (0.7%)	11 (1.4%)	<5
Angiotensin receptor blockers	65 (3.3%)	<5	61 (5.1%)
Beta-blockers	47 (2.4%)	14 (1.8%)	33 (2.8%)
Calcium channel blockers	99 (5.0%)	8 (1.0%)	91 (7.7%) †

† significant (*p* < 0.05) difference between females and males. Significance identified using *t*-test or chi-square with Yates’s correction as appropriate. Notes: ACE = Angiotensin converting enzyme. Individual cells with frequencies of <5 are compressed. The sum of reports from the United States and Japan does not equal the total reports, as reports from other countries (*N* = 20) are not reported due to lower event numbers per cell. The percentages reported are the column percentages taken from the total of either the overall (*N* = 1995), United States (*N* = 787), or Japan (*N* = 1188).

**Table 2:** Demographic characteristics of case reports with romosozumab (*N* = 1995), stratified by cardiovascular events.

	Non-Cardiovascular	MACE	Other Cardiovascular	Any Cardiovascular *
	1740	206	58	255
Sex				
Female	1323 (76.0%)	159 (77.2%) †	43 (74.1%)	195 (76.5%)
Male	143 (8.2%)	30 (14.6%) †	5 (8.6%)	34 (13.3%)
Unknown	274 (15.7%)	17 (8.3%) †	10 (17.2%)	26 (10.2%)
Age				
Mean (SD)	76.4 (9.9)	81.2 (9.5) †	79.0 (7.3)	81.1 (9.9)
18–59	41 (2.3%)	5 (2.4%)	--	5 (2.0%)
60–69	165 (9.5%)	6 (2.9%) †	4 (6.9%)	10 (3.9%)
70–79	324 (18.6%)	53 (25.7%) †	12 (20.7%)	61 (23.9%)
80+	335 (19.3%)	98 (47.6%) †	20 (34.5%)	116 (45.5%)
Unknown age	875 (50.3%)	44 (21.4%) †	22 (37.9%)	63 (24.7%)
Region of reporting				
United States	731 (42.0%)	41 (19.9%) †	16 (27.6%)	56 (22.0%)
Japan	990 (56.9%)	164 (79.6%) †	42 (72.4%)	198 (77.6%)
Other	19 (1.1%)	<5	--	<5
Seriousness				
Death	117 (6.7%)	54 (26.2%) †	7 (12.1%)	59 (23.1%)
Hospitalized or Required Intervention	526 (30.2%)	117 (56.8%) †	21 (36.2%)	134 (52.5%)
Life Threatening	19 (1.1%)	24 (11.7%) †	6 (10.3%)	29 (11.4%)
Disabled	10 (0.6%)	12 (5.8%) †	--	12 (4.7%)
Other co-reported cardiovascular drugs				
Anticoagulants	21 (1.2%)	13 (6.3%) †	4 (6.9%)	17 (6.7%)
Antiplatelets	34 (2.0%)	22 (10.7%) †	8 (13.8%)	26 (10.2%)
ACE inhibitors	9 (0.5%)	<5	<5	5 (2.0%)
Angiotensin receptor blockers	40 (2.3%)	22 (10.7%) †	5 (8.6%)	25 (9.8%)
Beta-blockers	25 (1.4%)	20 (9.7%) †	<5	22 (8.6%)
Calcium channel blockers	60 (3.4%)	34 (16.5%) †	6 (10.3%)	39 (15.3%)

* Any cardiovascular event is identified as those with a MACE and/or other cardiovascular outcomes. † significant (*p* < 0.05) difference between MACE and non-cardiovascular outcomes. Significance identified using *t*-test or chi-square with Yates’s correction as appropriate. Abbreviations: ACE = Angiotensin converting enzyme, MACE = major cardiovascular event. Notes: MACE is defined as the occurrence of myocardial infarction, cardiac death, or stroke. Other cardiovascular events are defined as the occurrence of coronary artery disease, thrombosis, bleeding, or cardiac disorders. Outcomes defined according to the Medical Dictionary for Regulatory Activities (MedDRA^®^) preferred terms (see Supplementary Table S2). The total number of case reports is the sum of no cardiovascular events (*N* = 1740) and any cardiovascular event (*N* = 255). As it is possible that a case may have both a MACE and another cardiovascular event reported, thus, these groups are not mutually exclusive. The seriousness criteria do not sum to the column totals as only major outcomes are reported, and groups are not mutually exclusive.

**Table 3:** Disproportionality analysis for the outcomes of interest, comparing events with romosozumab to events with all other drugs in the Food and Drug Administration Adverse Event Reporting System (FAERS) between 01 January 2019 and 31 December 2020.

	Romosozumab	Romosozumab	All other Drugs	All other Drugs			
	Event	No Event	Event	No Event	ROR (95% CI)	IC	IC_025_
MACE	206	1789	84,723	2,996,511	4.07 (2.39–6.93)	1.90	1.67
Myocardial infarction	42	1953	21,253	3,059,981	3.10 (1.43–6.72)	1.57	1.06
Stroke	84	1911	38,489	3,042,745	3.47 (1.81–6.69)	1.73	1.37
Cardiovascular death	86	1909	28,070	3,053,164	4.90 (2.55–9.40)	2.21	1.85
Other cardiovascular event	58	1937	56,239	3,024,995	1.61 (0.79–3.29)	0.66	0.23
General cardiac events	16	1979	16,880	3,064,354	1.47 (0.55–3.92)	0.53	−0.31
Bleeding	19	1976	20,699	3,060,535	1.42 (0.55–3.64)	0.49	−0.28
Thrombosis	23	1972	19,753	3,061,481	1.81 (0.74–4.44)	0.82	0.12

Abbreviations: ROR (reporting odds ratio), CI (confidence interval), IC (information component), IC025 (lower bound credibility interval for the IC). The outcomes of interest were identified using single or multiple preferred terms (PT) according to the Medical Dictionary for Regulatory Activities (MedDRA). A complete list can be found in the Supplementary Materials Table S1.

## Data Availability

The data are publicly available for download from the US FDA Adverse Event Reporting System (FAERS) Quarterly Files at: https://fis.fda.gov/extensions/FPD-QDE-FAERS/FPD-QDE-FAERS.html (last accessed: 8 February 2021).

## Supplementary Materials

The following are available online at https://www.mdpi.com/article/10.3390/jcm10081660/s1, Figure S1 Number of major cardiovascular events (MACE) reported in Japan by month and year, Table S1: Overview of outcome classification according to the Medical Dictionary for Regulatory Activities (MedDRA) preferred terms, Table S2: Overview of drug classification to identify co-reported cardiovascular drugs as either suspect, concomitant, or interacting, Table S3: Demographic characteristics of individual case safety reports with romosozumab (N = 1995), stratified by sex. Table S4: Disproportionality analysis of outcomes of interest among cases with a reported age of 50 years or older. Table S5: Disproportionality analysis of all outcomes of interest, stratified by case sex and reporting region.

## Appendix A

	Event of interest	All other events
Romosozumab	a	b
All other drugs in FAERS	c	d
Information component (IC): $IC=log_{2}\left(\left(N_{obs}+0.5\right)/\left(N_{exp}+0.5\right)\right)$ $N_{obs}=a$ $N_{exp}=\left((a+b\right)*\left(a+c\right))/\left(a+b+c+d\right)$ $IC_{025}=IC-3.3*{\left(N_{obs}+0.5\right)}^{-\frac{1}{2}}-2*\;{\left(N_{obs}+0.5\right)}^{-3/2}$	Reporting odds ratio (ROR): $ROR=\;\frac{a/b}{c/d}$ $SE_{\ln ROR}=\sqrt{\frac{1}{a}+\frac{1}{b}+\frac{1}{c}+\frac{1}{d}}$ $95\%\;CI_{ROR}=ROR\;*\;e^{\left(†1.96*\;SE_{\left(\ln ROR\right)}\right)}$	
**Equation 1.** Formulas for disproportionality measures. *Abbreviations: FAERS: Food and Drug Administration Adverse Event Reporting System, obs: observed, exp: expected, CI: confidence interaval*.		
