# Supplementary material for: Cardiovascular Safety Profile of Romosozumab: A Pharmacovigilance Analysis of the US Food and Drug Administration Adverse Event Reporting System (FAERS)
Source: J Clin Med. 2021 Apr 13;10(8):1660. doi: 10.3390/jcm10081660 (PMC8070537; doi:10.3390/jcm10081660)
Supplement: Supplementary file 1 [file jcm-10-01660-s001.pdf]

Supplementary Material

Figure S1 Number of major cardiovascular events (MACE) reported in Japan by month and year.

The solid vertical line represents the date of the investigation by the Japanese Pharmaceutical and Medical Devices Agency (PMDA) recommending a change to the warning listed in the romosozumab package insert. The y-axis represents the total number of reports submitted to the Food and Drug Administration Adverse Event Reporting System (FAERS) database. For analysis, the first FDA report date was selected for a given individual case safety report identification number.

Table S1 Overview of outcome classification according to the Medical Dictionary for Regulatory Activities (MedDRA) preferred terms

Table S2 Overview of drug classification to identify co-reported cardiovascular drugs as either suspect, concomitant, or interacting

Table S3 Demographic characteristics of individual case safety reports with romosozumab (N = 1995), stratified by sex

Table S4 Disproportionality analysis of outcomes of interest among cases with a reported age of 50 years or older

Table S5. Disproportionality analysis of all outcomes of interest, stratified by case sex and reporting region

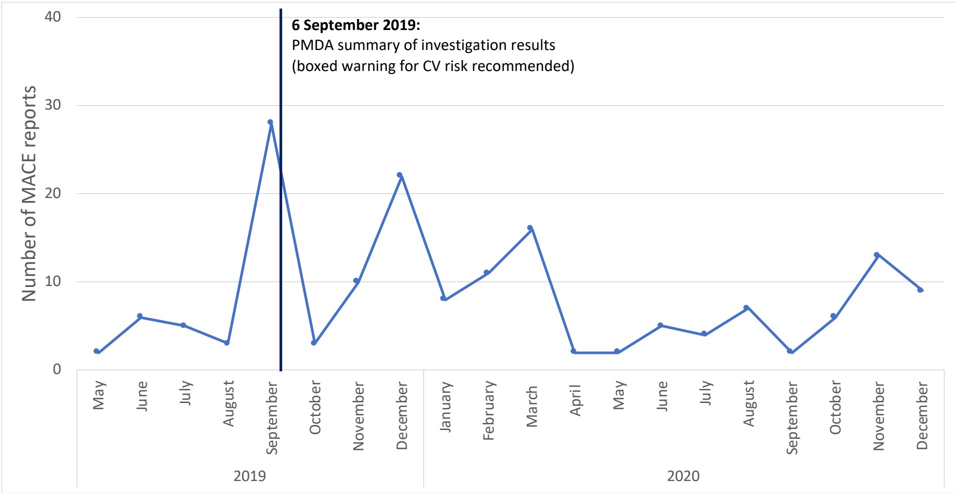

Figure S1 Number of major cardiovascular events (MACE) reported in Japan by month and year.

The solid vertical line represents the date of the investigation by the Japanese Pharmaceutical and Medical Devices Agency (PMDA) recommending a change to the warning listed in the romosozumab package insert. The y-axis represents the total number of reports submitted to the Food and Drug Administration Adverse Event Reporting System (FAERS) database. For analysis, the first FDA report date was selected for a given individual case safety report identification number.

Table S1 Overview of outcome classification for cardiovascular events, according to the Medical Dictionary for Regulatory Activities (MedDRA) preferred terms

High Level Term	Preferred Term	Study Classification	Study Group
Cardiac disorders	Cardiac Failure	Cardiovascular death	MACE
Cardiac disorders	Cardio-Respiratory Arrest	Cardiovascular death	MACE
Cardiac disorders	Cardiac Arrest	Cardiovascular death	MACE
Cardiac disorders	Cardiac Failure Acute	Cardiovascular death	MACE
Cardiac disorders	Myocardial Infarction	Myocardial Infarction	MACE
Cardiac disorders	Myocardial Ischaemia	Myocardial Infarction	MACE
Cardiac disorders	Acute Myocardial Infarction	Myocardial Infarction	MACE
Cardiac disorders	Coronary Artery Stenosis	Myocardial Infarction	MACE
Cardiac disorders	Acute Coronary Syndrome	Myocardial Infarction	MACE
Cardiac disorders	Coronary Artery Occlusion	Myocardial Infarction	MACE
Cardiac disorders	Coronary Artery Thrombosis	Myocardial Infarction	MACE
Cardiac disorders	Cardiac Disorder	General	Other Cardiovascular
Cardiac disorders	Cardiovascular disorder	General	Other Cardiovascular
Cardiac disorders	Coronary Artery Disease	General	Other Cardiovascular
Nervous System Disorder	Cerebral Haemorrhage	Stoke	MACE
Nervous System Disorder	Cerebral Infarction	Stoke	MACE
Nervous System Disorder	Cerebellar Haemorrhage	Stoke	MACE
Nervous System Disorder	Cerebellar Infarction	Stoke	MACE
Nervous System Disorder	Cerebrovascular Accident	Stoke	MACE
Nervous System Disorder	Transient Ischaemic Attack	Stoke	MACE
Nervous System Disorder	Ischaemic Stroke	Stoke	MACE
Nervous System Disorder	Haemorrhagic Stroke	Stoke	MACE
Nervous System Disorder	Ischaemic Cerebral Infarction	Stoke	MACE
Nervous System Disorder	Intracranial Aneurysm	Stoke	MACE
Nervous System Disorder	Haemorrhage Intracranial	Stoke	MACE
Nervous System Disorder	Lacunar Infarction	Stoke	MACE
Nervous System Disorder	Ruptured Cerebral Aneurysm	Stoke	MACE
Nervous System Disorder	Putamen Haemorrhage	Stoke	MACE
Nervous System Disorder	Subarachnoid Haemorrhage	Stoke	MACE
Nervous System Disorder	Embolic Stroke	Stoke	MACE
Nervous System Disorder	Thalamus Haemorrhage	Stoke	MACE
Nervous System Disorder	Central Nervous System Haemorrhage	Stoke	MACE
Nervous System Disorder	Basel Ganglia Haemorrhage	Stoke	MACE
Nervous System Disorder	Basel Ganglia Infarction	Stoke	MACE
Vascular Disorder	Aortic Dissection	Bleeding	Other cardiovascular
Vascular Disorder	Internal Haemorrhage	Bleeding	Other cardiovascular
Vascular Disorder	Aortic Aneurysm	Bleeding	Other cardiovascular
Vascular Disorder	Haemorrhage	Bleeding	Other cardiovascular
Vascular Disorder	Aortic Aneurysm Rupture	Bleeding	Other cardiovascular
Vascular Disorder	Shock Haemorrhage	Bleeding	Other cardiovascular
Vascular Disorder	Deep Vein Thrombosis	Thrombosis	Other cardiovascular
Vascular Disorder	Thrombosis	Thrombosis	Other cardiovascular
Vascular Disorder	Peripheral Artery Occlusion	Thrombosis	Other cardiovascular
Vascular Disorder	Peripheral Arterial Occlusive Disease	Thrombosis	Other cardiovascular
Vascular Disorder	Venous Thrombosis Limb	Thrombosis	Other cardiovascular
Vascular Disorder	Atheroembolism	Thrombosis	Other cardiovascular
Vascular Disorder	Embolism Venous	Thrombosis	Other cardiovascular
Vascular Disorder	Pelvic Venous Thrombosis	Thrombosis	Other cardiovascular
Vascular Disorder	Vascular Occlusion	Thrombosis	Other cardiovascular

Table S2 Overview of drug classifications to identify co-reported cardiovascular drugs as either suspect, concomitant, or interacting

**Anticoagulants**	**Angiotensin receptor blockers**
dabigatran	Candesartan
enoxaparin	Irbesartan
apixaban	Olmesartan
rivaroxaban	Losartan
endoxaban	Valsartan
heparin	Telmisartan
warfarin	Eprosartan
**Antiplatelets**	**Beta Blockers**
Aspirin	Atenolol
Clopidogrel	Acebutolol
Dipyridamole	Bisoprolol
Prasugrel	Carvedilol
Ticlopidine	Celiprolol
Ticagrelor	Esmolol
	Labetalol
	Metoprolol
	Propranolol
	Sotalol
**Angiotensin converting enzyme inhibitors**	**Calcium Channel Blockers**
Benazepril	Amlodipine
Captopril	Diltiazem
Cilazapril	Felodipine
Enalapril	Isradipine
Fosinopril	Nifedipine
Lisinopril	Verapamil
Moexioril	Nicardipine
Perinopril	Nisoldipine
Quinapril	
Riamipril	
Trandolapril	

Table S3 Demographic characteristics of individual case safety reports with romosozumab (N=1,995), stratified by sex

**Characteristic**	**Total**	**Male**	**Female**	**Unknown**
	1995	177 (8.9%)	1518 (76.1%)	300 (15.0%)
Age				
Mean (SD)	77.0 (10.2)	79.4 (9.4)†	76.7 (10.1)	80.9 (9.6)
18 – 39	<5	--	<5	--
40 – 49	7 (0.7%)	--	7 (0.8%)	--
50 – 59	36 (3.4%)	6 (3.4%)	30 (3.2%)	--
60 – 69	175 (16.6%)	12 (6.8%)†	162 (17.5%)	<5
70 – 79	385 (36.4%)	37 (20.9%)	344 (25.1%)	<5
80+	451 (42.7%)	67 (37.9%)†	377 (30.8%)	7 (2.3%)
Unknown age	938 (47.0%)	55 (31.1%)†	595 (39.2%)	288 (96.0%)
Region of reporting				
United States	787 (39.4%)	22 (12.4%)†	669 (44.0%)	96 (32.0%)
Japan	1188 (59.5%)	154 (87.0%)†	833 (54.9%)	201 (67.0%)
Other	20 (1.0%)	1 (0.6%)	16 (1.1%)	<5
Seriousness Criteria*				
Death	176 (8.8%)	36 (20.3%)†	122 (9.2%)	18 (6.0%)
Hospitalized or Required Intervention	660 (33.1%)	81 (45.8%)†	489 (32.2%)	90 (30.0%)
Life Threatening	48 (2.4%)	9 (5.1%)	36 (3.3%)	<5
Disabled	22 (1.1%)	7 (4.0%)†	15 (1.5%)	--
Outcomes of Interest				
Major Cardiovascular Event	206 (10.3%)	30 (16.9%)†	159 (10.5%)	17 (5.7%)
Myocardial Infarction	42 (2.1%)	10 (5.6%)†	29 (1.9%)	<5
Stroke	84 (4.2%)	6 (3.4%)	71 (4.7%)	7 (2.3%)
Cardiovascular Death	86 (4.3%)	14 (7.9%)†	64 (4.2%)	8 (2.7%)
Other Cardiovascular Event	58 (2.9%)	5 (2.8%)	43 (2.8%)	10 (3.3%)
General cardiac events	16 (0.8%)	<5	8 (0.5%)	7 (2.3%)
Bleeding	19 (1.0%)	<5	14 (0.9%)	<5
Thrombosis	23 (1.2%)	<5	21 (1.4%)	<5
Other reported cardiovascular drugs				
Anticoagulants	38 (1.9%)	<5	32 (2.1%)	<5
Antiplatelets	60 (3.0%)	13 (7.3%)†	46 (3.0%)	<5
Angiotensin converting enzyme inhibitors	14 (0.7%)	--	14 (0.9%)	--
Angiotensin receptor blockers	65 (3.3%)	11 (6.2%)	54 (3.6%)	--
Beta-blockers	47 (2.4%)	10 (5.6%)†	37 (2.4%)	--
Calcium channel blockers	99 (5.0%)	18 (10.2%)†	80 (5.3%)	<5

*Seriousness criteria do not sum to total as only the major outcomes are reported here and categories are not mutually exclusive.
† significant ($p<0.05$) difference between females and males. Significance identified using t-test or chi-square with Yates's correction as appropriate.
Notes: Individual cells with frequencies of <5 are compressed.

Table S4 Disproportionality analysis of outcomes of interest among cases with a reported age of 50 years or older

	Romosozumab Event	Romosozumab No event	All other drugs Event	All other drugs No event	ROR (95% CI)	IC	IC025
MACE	159	888	47,336	1,070,507	4.05 (2.28-7.20)	1.83	1.57
Myocardial infarction	34	1,013	12,100	1,105,743	3.07 (1.35-6.96)	1.54	0.97
Stroke	66	981	21,707	1,096,136	3.40 (1.69-6.84)	1.67	1.26
Cardiovascular death	64	983	15,292	1,102,551	4.69 (2.32-9.50)	2.12	1.70
Other cardiovascular event	36	1,011	24,509	1,093,334	1.59 (0.71-3.56)	0.64	0.08
General cardiovascular events	<5	1,043	7,054	1,110,789	0.60 (0.15-2.42)	-0.66	-2.42
Bleeding	14	1,033	9,041	1,108,802	1.66 (0.60-4.60)	0.69	-0.21
Thrombosis	18	1,029	8,949	1,108,894	2.17 (0.83-5.64)	1.06	0.26

Abbreviations: ROR (Reporting Odds Ratio), CI (Confidence Interval), IC (Information Component, IC025 (Lower bound credibility interval for the IC).

Note: The outcomes of interest were identified using single or multiple preferred terms (PT) according to the Medical Dictionary for Regulatory Activities (MedDRA), a complete list can be found in the supplementary material, Table S1. Analysis only includes cases where age was recorded (non-missing) and where age was age 50 years or older (Figure 1).

Table S5 Disproportionality analysis of all outcomes of interest, stratified by case sex and reporting region

		Romosozumab Event	Romosozumab No event	All other drugs Event	All other drugs No event	ROR (95% CI)	IC	IC_{025}
MACE	All cases	206	1,789	84,723	2,996,511	4.07 (2.39-6.93)	1.90	1.67
	Women	159	1,359	37,081	1,575,209	4.97 (2.82-8.77)	2.17	1.90
	Men	30	147	38,745	989,627	5.21 (2.17-12.53)	2.09	1.48
	United States	41	1,147	39,295	2,012,447	1.83 (0.84-4.00)	0.84	0.32
	Japan	164	623	5,788	78,169	3.56 (1.98-6.38)	1.56	1.30
Myocardial infarction	All cases	42	1,953	21,253	3,059,981	3.10 (1.43-6.72)	1.57	1.06
	Women	29	1,489	2,404	1,609,886	13.04 (5.57-30.55)	3.40	2.78
	Men	10	167	3,292	1,025,080	18.65 (6.09-57.10)	3.30	2.22
	United States	13	1,175	10,062	2,041,680	2.24 (0.80-6.32)	1.09	0.15
	Japan	28	759	695	83,262	4.42 (1.86-10.53)	1.98	1.35
Stroke	All cases	84	1,911	38,489	3,042,745	3.47 (1.81-6.69)	1.73	1.37
	Women	71	1,447	5,409	1,606,881	14.58 (7.34-28.93)	3.66	3.27
	Men	6	171	5,079	1,023,293	7.07 (2.00-25.01)	2.24	0.83
	United States	27	1,161	19,022	2,032,720	2.49 (1.05-5.90)	1.25	0.61
	Japan	57	730	3,101	80,856	2.04 (0.98-4.22)	0.95	0.51
Cardiovascular death	All cases	86	1,909	28,070	3,053,164	4.90 (2.55-9.40)	2.21	1.85
	Women	64	1,454	3,430	1,608,860	20.65 (10.22-41.73)	4.09	3.68
	Men	14	163	3,986	1,024,386	22.07 (7.84-62.15)	3.61	2.71
	United States	<5	1,185	11,540	2,040,202	0.45 (0.10-1.99)	-1.04	-3.11
	Japan	83	704	2,156	81,801	4.47 (2.28-8.77)	1.97	1.61
Other cardiovascular event	All cases	58	1,937	56,239	3,024,995	1.61 (0.79-3.29)	0.66	0.23
	Women	43	1,475	26,223	1,586,067	1.76 (0.82-3.81)	0.79	0.28
	Men	5	172	23,171	1,005,201	1.26 (0.34-4.72)	0.29	-1.27
	United States	16	1,172	34,156	2,017,586	0.81 (0.30-2.16)	-0.30	-1.14
	Japan	42	745	1,638	82,319	2.83 (1.29-6.21)	1.40	0.89
General cardiovascular events	All cases	16	1,979	16,880	3,064,354	1.47 (0.55-3.92)	0.53	-0.31
	Women	8	1,510	1,678	1,610,612	5.09 (1.58-16.36)	2.03	0.81
	Men	<5	176	1,608	1,026,764	3.63 (0.51-25.84)	0.95	-2.83
	United States	6	1,182	10,160	2,041,582	1.02 (0.29-3.57)	0.03	-1.39
	Japan	10	777	207	83,750	5.21 (1.70-15.94)	2.06	0.98
Bleeding	All cases	19	1,976	20,699	3,060,535	1.42 (0.55-3.64)	0.49	-0.28
	Women	14	1,504	1,603	1,610,687	9.35 (3.38-25.88)	2.84	1.94
	Men	<5	174	1,652	1,026,720	10.72 (2.40-47.85)	2.16	0.09
	United States	<5	1,188	13,035	2,038,707		-4.01	-14.33
	Japan	19	768	854	83,103	2.41 (0.93-6.22)	1.18	0.41
Thrombosis	All cases	23	1,972	19,753	3,061,481	1.81 (0.74-4.44)	0.82	0.12
	Women	21	1,497	3,106	1,609,184	7.27 (2.90-18.24)	2.64	1.91
	Men	<5	176	2,281	1,026,091	2.56 (0.36-18.20)	0.75	-3.03
	United States	10	1,178	11,555	2,040,187	1.50 (0.50-4.52)	0.55	-0.53
	Japan	13	774	591	83,366	2.37 (0.84-6.72)	1.14	0.21

Abbreviations: ROR (Reporting Odds Ratio), CI (Confidence Interval), IC (Information Component, IC025 (Lower bound credibility interval for the IC).
Note: The outcomes of interest were identified using single or multiple preferred terms (PT) according to the Medical Dictionary for Regulatory Activities (MedDRA), a complete list can be found in the supplementary material, Table S1.
